# Batch Adsorption of Orange II Dye on a New Green Hydrogel—Study on Working Parameters and Process Enhancement

**DOI:** 10.3390/gels11010079

**Published:** 2025-01-20

**Authors:** Andrei-Ionuț Simion, Cristina-Gabriela Grigoraș, Lidia Favier

**Affiliations:** 1 Department of Food and Chemical Engineering, Faculty of Engineering, “Vasile Alecsandri” University of Bacău, Calea Mărăşeşti 157, 600115 Bacau, Romania; asimion@ub.ro; 2 Ecole Nationale Supérieure de Chimie de Rennes, Univ. Rennes, CNRS, UMR 6226, CEDEX 7, 35708 Rennes, France; lidia.favier@ensc-rennes.fr

**Keywords:** adsorption, cherry stones, desorption, equilibrium isotherm, hydrogel, kinetic study, Orange II, sodium alginate, thermodynamic study, ultrasound

## Abstract

A new green hydrogel consisting of cherry stone (CS) powder and sodium alginate (SA) was synthesized through physical crosslinking. The product had a mean diameter of 3.95 mm, a moisture content of 92.28%, a bulk density of 0.58 g/cm^3^, and a swelling ratio of 45.10%. The analyses of its morphological structure and functional groups by scanning electron microscopy (SEM) and Fourier-transform infrared spectroscopy (FTIR) showed the successful entrapping of the CS in the SA polymeric matrix. The viability of the prepared hydrogel as adsorbent was tested towards Orange II (OII) anionic dye. The influence of the *p*H, adsorbent amount, contact time, and initial dye concentration was evaluated. Then, the impact of three accelerating factors (stirring speed, ultrasound exposure duration, and temperature) on the OII retention was investigated. The highest recorded removal efficiency and adsorption capacity were 82.20% and 6.84 mg/g, respectively. The adsorption followed Elovich and pseudo-second-order kinetics, was adequately described by Freundlich and Khan isotherms, and can be defined as spontaneous, endothermic, and random. The experiments confirmed that the obtained hydrogel can be used acceptably for at least two consecutive cycles, sustaining its effectiveness in water decontamination.

## 1. Introduction

Adsorption has been shown to be one of the most efficient and adaptable strategies for wastewater management. Because of its ease of use and effectiveness, it addresses the prevalent concern of the industrial sector and individuals regarding access to clean water. The process is based on the retention of water pollutants such as personal care products [1,2], pharmaceuticals [3,4,5], pesticides [6,7], dyes [8,9,10], and heavy metals [11,12] on the surface of different materials. Among these materials, the activated carbon obtained through thermal, chemical, or thermochemical activation of a precursor definitively stands out. Ruszczyk et al. [13] investigated the efficiency of activated carbon prepared from coconut shells in the removal of triclosan from water. According to their results, more than 85% of the contaminant was retained. Murti et al. [14] analyzed the potential of *Cassia fistula* as raw material to produce activated carbon and concluded that the prepared product was able to remove isoniazid from water. Zhu et al. [15] used available literature data to build machine learning algorithms for constructing and evaluating predictive models about the adsorption of pharmaceuticals and personal care products on activated carbon and biochars. The study published by Zgolli et al. [16] revealed the adequacy of activated carbon obtained from prickly pear seeds for the adsorption of deltamethrin. A low-cost date palm fiber activated carbon was prepared by Melliti et al. [17]. Their tests on the adsorption of two heavy metals indicated that the adsorbent removed more than 95% of Pb(II) and more than 80% of Cu(II). The large variety of production sources, the high porosity, the increased surface area, and the surface functional groups of the activated carbon, facilitate the adsorption of emerging water compounds. Nevertheless, the production process is expensive and time-consuming. The involved methods imply drying, carbonization, and activation with steam or carbon dioxide at elevated temperatures (in the case of physical activation); the use of chemical activation agents such as acids, bases, or salts employed to dehydrate and oxidize the raw material followed by exposure at high temperatures (in the case of chemical activation); or application of a preliminary treatment with a chemical reagent followed by exposure to elevated temperatures (in the case of thermochemical activation) [18]. Regeneration difficulties and disposal concerns also contribute to the drawbacks of using activated carbon. Subsequently, the development of other adsorbents able to remove water pollutants continues to expand an increasing interest being directed to biodegradable polymeric materials with three-dimensional structures and a hydrophilic nature, known as hydrogels [19,20]. Various classification criteria divide these adsorbents into different types. Thus, depending on the type of source, they can be natural or synthetic; depending on their structure, they can be homopolymeric or copolymeric; based on the applied crosslinking process, they are separated into physical and chemical while, based on the charge type, they can be identified as anionic, cationic, or amphoteric [21]. Hydrogels are reported as possessing an important affinity for different unwanted compounds in water. Kim et al. [22] designed a multifunctional copolymer hydrogel that efficiently removed fluorinated alkyl substances from aqueous media. A promising anionic hydrogel composed of date palm leaves and cellulose nanofiber was obtained by Fadhillah and his collaborators. Tests carried out on the adsorption of methylene blue revealed that, in the optimized working conditions (*p*H 6, 60 °C), an adsorption capacity of 5 mg/g was reached [23]. Al-Harby et al. studied the retention of copper ions from water on hydrogels prepared from chitosan crosslinked with trimellitic anhydride isothiocyanate. Their results showed that, at *p*H 6 and 25 °C, 15 mg of adsorbent removed more than 95% of the metallic ions [24]. Guo et al. demonstrated in one of their research papers that chitosan-modified bentonite hydrogels were able to eliminate tetracycline from water [25]. Lin et al. [26] synthesized an alginate–metal–organic framework composite hydrogel modified by polyethylenimine which adsorbed 68.75 mg/g of phosphate from water. After five consecutive cycles, its adsorption rate was 99%. Azam et al. evaluated the ability of a hydrogel composite made of nonwoven fabric with alginate, gum arabic, and xanthan gum to adsorb lead ions from aqueous solutions and they stated a removal efficiency higher than 88% after 60 min of contact [27].

The particularity of the current research is highlighted by its recommendation of a hydrogel formulated through a technique consisting solely of embedding an underappreciated industrial byproduct within a natural polymeric matrix, with the aim of obtaining a material that is ready for immediate use as adsorbent for water decontamination.

CS and SA were used as raw materials and physical crosslinking was employed as the synthesis method. The physical properties, the surface morphology, the functional groups, and the point of zero charge of the resulting hydrogel (CSSA) were established. OII, an azo dye, with high toxicity and difficult degradation through conventional water treatment methods [28], was selected as the test molecule. The influence of parameters such as *p*H, adsorbent amount, contact time, and initial dye concentration was assessed to find the adequate working conditions. The effect of stirring, ultrasound exposure, and temperature was appraised in terms of process intensification settings. Different kinetic and equilibrium isotherm models were applied to the experimentally acquired data and, along with the thermodynamic analysis, were used in the exploration of the model molecule adsorption behavior on the synthesized hydrogel. At the end, the reusability of the prepared material was assessed in order to evaluate the possibility of using it for multiple consecutive adsorption cycles.

## 2. Results and Discussion

### 2.1. Hydrogel Preparation and Characterization

The diversity of the contaminants encountered in the aquatic environment is challenging. Since it can be performed with remarkable results, adsorption is considered an attractive alternative to overcome the downsides of the classical treatment technologies.

In the light of the ongoing attempts to create different adsorbents, the first step of the current study aimed to synthesize a new, green, low-cost material. CS—a byproduct from the fruit-processing industry—was chosen as a precursor. The decision was based on the fact that it is often discarded, causing significant environmental concerns [29], and is rapidly becoming available at a very reduced price. Our preliminary experiments revealed on the one hand that, in its natural form, CS is not able to retain pollutants from water and on the other hand that its transformation in powder led to the solubilization of various constituents when the contact with water was ensured. Hence the need to protect the precursor without diminishing its potential to retain water contaminants. Sodium alginate (a natural anionic biopolymer that is inexpensive, nontoxic, and biodegradable) was considered appropriate for this purpose since it was reported elsewhere as suitable in the production of other adsorbents [30,31,32].

A mixture of CS and SA was dripped into a calcium chloride solution. CSSA gel beads with a round, regular, uniform shape and a yellowish white shade formed immediately through physical ionic crosslinking occurring between the carboxylate groups of SA and the calcium ions [21]. The beads had a mean diameter of 3.95 mm, a moisture content of 92.28%, and a bulk density of 0.58 g/cm^3^. The swelling ratio was 45.10% at *p*H 3, 55.77% at *p*H 5, 50.01% at *p*H 7, 44.90% at *p*H 9, and 42.00% at *p*H 11. The swelling ratio increased in acidic media and decreased in neutral and alkaline environments, sustaining the *p*H impact on the CSSA hydrogel. This fact is contrary to the observation made by Muthumari et al. who revealed that SA shrank in acidic media and the swelling ability of beads diminished [33]. A possible explanation for this divergence could be attributed to the composition of the hydrogel. Besides SA, CSSA also contains an important amount of CS powder which could influence the swelling index and affect the ability of the hydrogel to retain water. This behavior was also detected by Wu et al. [34] who fabricated a Huangshui alginate hydrogel and noted that the bead swelling increased at *p*H 2 to 4 and decreased at higher *p*H values.

**Figure 1:**
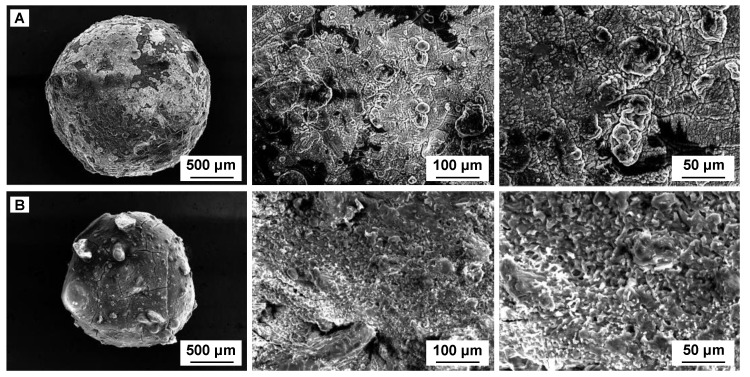
SEM images of the CSSA hydrogel before (**A**) and after (**B**) OII adsorption.

The morphological analysis of the CSSA hydrogel, shown in SEM photographs of Figure 1, displays a smooth, homogeneous surface and a porous internal nature. Changes appearing after OII adsorption are visible and suggest that the material is appropriate for the removal of the considered target molecule from the water matrix.

**Figure 2:**
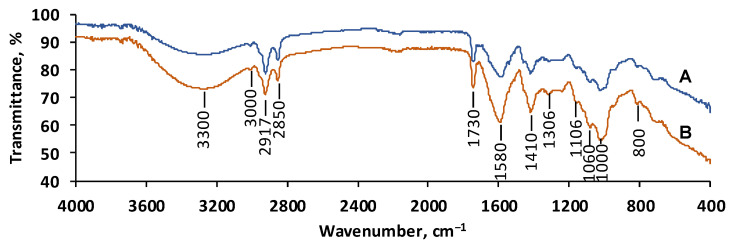
FTIR spectra of CSSA hydrogel before (**A**) and after (**B**) OII adsorption.

The functional groups of the CSSA hydrogel are pictured in Figure 2. In the FTIR spectrum recorded before OII adsorption, the broad peak detected around 3400 cm^−1^ to 3200 cm^−1^ is specific to the O–H stretching vibrations while peaks at approximately 2900 cm^−1^ correspond to the C–H bonds from the methyl and methylene groups existing in the prepared hydrogel [35,36]. Asymmetric and symmetric stretching vibrations of carboxylate groups can be assigned at 1600 cm^−1^ and 1400 cm^−1^ while C–O stretching vibration is detected at around 1100 cm^−1^ [37]. Peaks at 1100 cm^−1^ and 1060 cm^−1^ are ascribed to C–N and C–O vibrations, respectively.

The intensity differences in the peaks (especially those at 3400 cm^−1^, 1200 cm^−1^, and 1100 cm^−1^) exhibited in the spectrum obtained after OII adsorption are due to symmetric vibrations of the O–H, the –O–S–(O_2_) group, and –N=N stretching vibrations, being suggestive of the interactions occurring between the dye molecule and the CSSA hydrogel and thus confirming the adsorption of the target compound on the synthesized material.

### 2.2. Effect of Working Parameters on the OII Adsorption

#### 2.2.1. Point of Zero Charge and the Effect of *p*H

**Figure 3:**
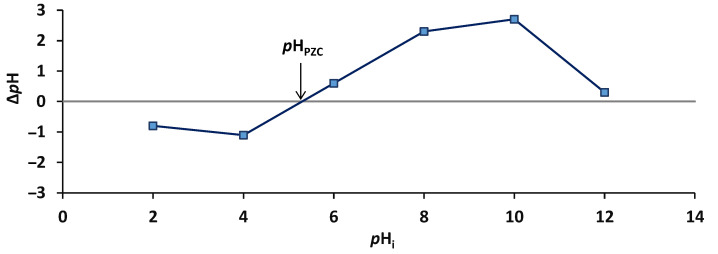
*p*H_PZC_ of CSSA hydrogel.

The point of zero charge (*p*H_PZC_) affects the positive and negative charges of an adsorbent, the electrical charge of the OII, and its degree of ionization. When the working *p*H is set below the *p*H_PZC_, the adsorbent is considered to have a positively charged surface, and vice versa (when the working *p*H is above the *p*H_PZC_, the adsorbent surface is negatively charged). The *p*H_PZC_ of the CSSA hydrogel (Figure 3) was established at 5.2.

OII is an anionic dye and thus it will be retained by an adsorbent possessing positive charges which is the case of CSSA at *p*H inferior to *p*H_PZC_ when the proton excess favors the adsorption process and the electrostatic attraction and interaction between the adsorbate and the adsorbent are facilitated. Comparable data are disclosed by other reports in which the elimination of anionic dyes such as yellow 161, remazol red 5B, or tartrazine by adsorption on various adsorbents was studied [38,39,40].

**Figure 4:**
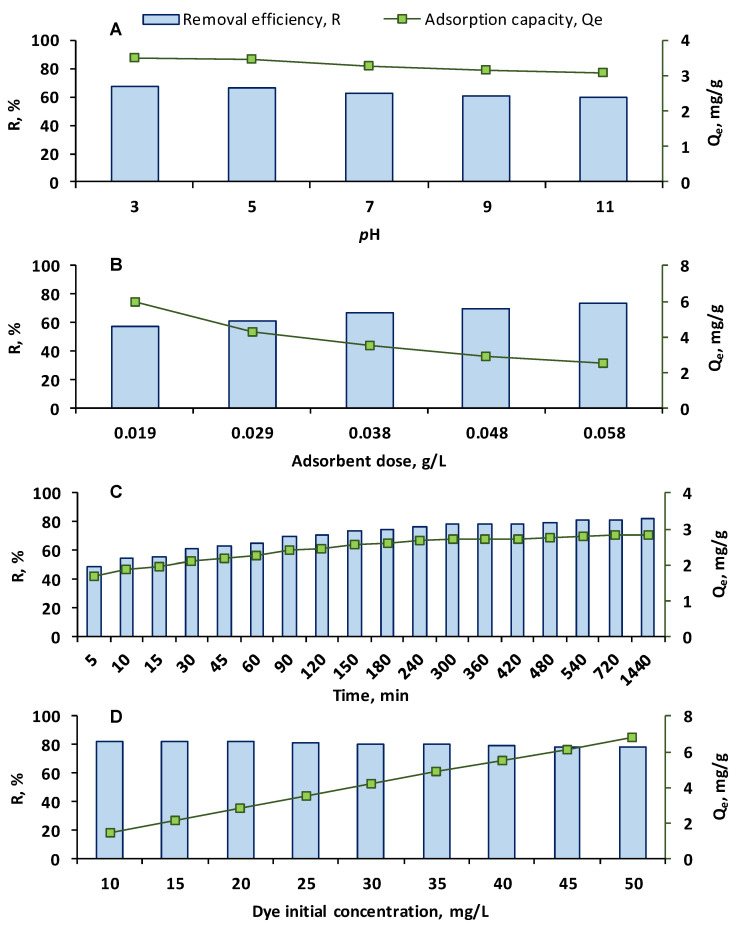
Influence of *p*H (**A**), adsorbent dose (**B**), time (**C**), and initial dye concentration (**D**) on the removal of OII from aqueous solution through adsorption on CSSA hydrogel (the experiments were carried out with 10 mL of OII, at room temperature, without stirring, the working conditions being as follows A: OII concentration—20 mg/L, *p*H—variable, adsorbent dose—0.038 g/L, contact time—300 min; B: OII concentration—20 mg/L, *p*H—3, adsorbent dose—variable, contact time—300 min; C: OII concentration—20 mg/L, *p*H—3, adsorbent dose—0.058 g/L, contact time—variable; D: OII concentration—variable, *p*H—3, adsorbent dose—0.058 g/L, contact time—300 min).

In order to evaluate the impact of the *p*H on the OII adsorption, volumes of 10 mL of dye solution with a concentration of 20 mg/L and five different *p*H values (3, 5, 7, 9, 11) were put in contact with 0.038 g/L CSSA for 300 min at room temperature. The highest removal efficiency (67.17%) and adsorption capacity (3.49 mg/g) were recorded at *p*H 3 (Figure 4A) while the lowest values were detected at *p*H 11, endorsing the idea described earlier that the CSSA hydrogel is sensitive to variations in the environmental *p*H. Keshmiri-Naqab et al. [41] prepared a granular-based bentonite from biowastes, tested its performance against OII adsorption, and found that the best results were observed at acidic *p*H and that an augmentation of *p*H over the *p*H_PZC_ (4.8) led to a reduction in the dye retention on the adsorbent. Perez-Calderon et al. conducted research on the adsorption of OII on an eco-friendly PVA–chitosan film and discovered that the recommended *p*H was also acidic (2.5). In this medium, the dye molecule exists in its doubly protonated and mono-protonated form, its sulfonate and hydroxyl groups being able to interact with the positive charges of the adsorbent [42].

#### 2.2.2. Effect of Adsorbent Dose

Another key factor of the adsorption process is represented by the adsorbent dose. Several experiments were carried out for 300 min at room temperature with different amounts of the prepared CSSA hydrogel added to 10 mL of OII solution with a concentration of 20 mg/L and *p*H 3. As can be noted from Figure 4B, when the adsorbent dose was 0.019 g/L, the removal rate was 57.75% and the adsorption capacity was 6.01 mg/g while an increase in CSSA dose to 0.058 g/L (retained as appropriate for continuing the OII adsorption studies) generated an increase in the removal efficiency up to 73.75% and a decrease in the adsorption capacity to 2.56 mg/g. This is in agreement with the fact that the amount of CSSA is correlated with the number of active sites available for the adsorption of OII. Amiri et al. [8] realized a study focused on the adsorption of three different dyes in single, binary, and ternary systems on starch nanocrystals. They highlighted that at a lower adsorbent dosage, the active sites are more quickly occupied by the target molecules and that, at a higher adsorbent dosage, the sites with lower energy are more easily occupied, resulting in a reduction in the adsorption capacity. In their study on the enhancement of OII adsorption on a copper-based metal–organic framework supporting a ZnO composite, Yardimci et al. showed that the adsorption efficiency rose when the adsorbent dosage increased from 0.01 g to 0.015 g and that, after this limit, the removal efficiency was less affected since the amount of dye in the aqueous solution was reduced [43]. Xu et al., who prepared an adsorbent based on bentonite modified with hydroxy-aluminium and cetyltrimethyl ammonium bromide and assessed its ability to remove OII from aqueous solution, remarked that an increase in the adsorbent quantity after a certain amount will not have an impact on the removal efficiency and will increase the process cost [44].

#### 2.2.3. Effect of Contact Time and Kinetic Studies

Contact time’s influence on the adsorption of OII on the synthesized hydrogel was investigated from 5 min to 1440 min at room temperature in an OII solution with 20 mg/L concentration, *p*H 3, and an adsorbent dose of 0.058 g/L. As illustrated in Figure 4C, the increase in the contact time was beneficial for the elimination of OII from water. After the first 5 min, the removal efficiency was 48.64% and the adsorption capacity was 1.69 mg/g. The values amplified to 81.64%, and 2.83 mg/g, respectively, after 720 min. The increase in time up to 1440 min led to a removal efficiency of 82.20% and an adsorption capacity of 2.85 mg/g. The reduced augmentation could be explicated by the fact that an equilibrium was reached, the advantages of a longer contact time being insufficient for the continuation of the adsorption. Rapid uptake followed by slower adsorption are reported by other researchers as well. Yilmazoglu et al. synthesized imidazolium-based ionic liquid modified montmorillonite materials for the adsorption of OII and revealed that the equilibrium was attained between 120 min and 180 min [45]. Xu et al. disclosed a more rapid kinetic (with a removal efficiency of more than 80% in only 30 min) for the adsorption of OII [44].

For a better understanding of the process, the experimental data were fitted with pseudo-first-order, pseudo-second-order, and Elovich kinetic models known to be adequate for describing the dye adsorption process [46]. The pseudo-first-order kinetic describes adsorption in heterogeneous systems and considers that the active sites are each occupied by an adsorbate molecule through physical adsorption. The pseudo-second-order model states that the chemisorption is the rate-limiting step and it is based on the interactions between the adsorbent and the adsorbate. The Elovich model considers chemisorption as the main mechanism governing the adsorption and the adsorbent surface as heterogeneous. It assumes an increase in the activation energy with time [47,48].

**Figure 5:**
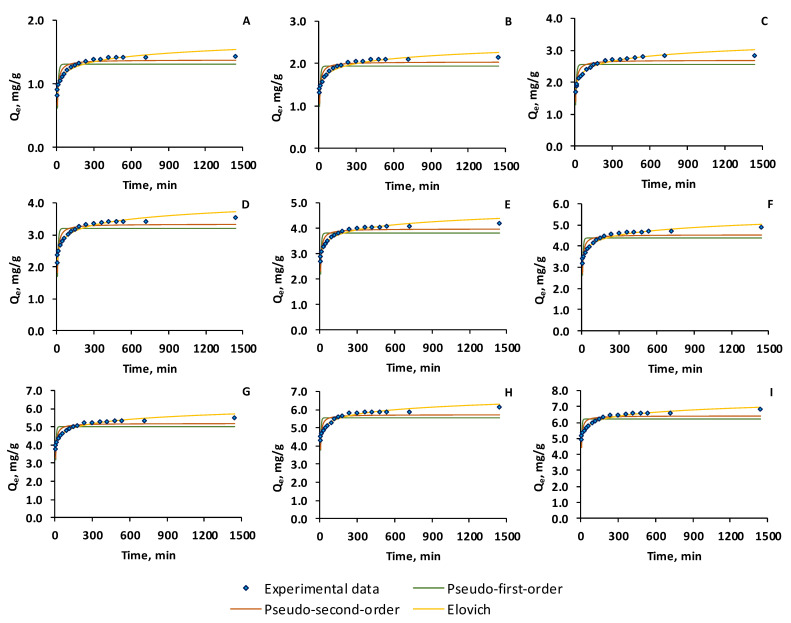
Kinetic models of the OII adsorption on CSSA hydrogel (initial OII concentration: (**A**) 10 mg/L, (**B**) 15 mg/L, (**C**) 20 mg/L, (**D**) 25 mg/L, (**E**) 30 mg/L, (**F**) 35 mg/L, (**G**) 40 mg/L, (**H**) 45 mg/L, (**I**) 50 mg/L) (the experiments were carried out with 10 mL of OII, at room temperature, without stirring, the working conditions being as follows: *p*H—3, adsorbent dose—0.058 g/L, contact time—variable).

Figure 5 shows the fitting curves of the adsorption capacity over time.

The kinetic parameters and the statistical error functions (root mean square error (RMSE), chi-squared test (χ^2^), Marquardt’s percent standard deviation (MPSD), hybrid fractional error function (HYBRID), coefficient of determination (R^2^), corrected Akaike information criterion (AICc), and Bayesian information criterion (BIC)) are given in Table 1. By comparing the fitting of the four kinetic models, it can be noted that the higher values of the correlation coefficients and the smallest values for the other error functions follow the sequence Elovich < pseudo-second order < pseudo-first order.

The analysis of the kinetic parameters led to the conclusion that the values of equilibrium adsorption capacity calculated with the pseudo-second-order model were closer to the experimental data than those obtained with the pseudo-first-order equation, suggesting that the adsorption of OII on the CSSA hydrogel occurs through chemisorption. The extent of surface coverage and activation energy for chemisorption of the Elovich model decreased with the augmentation of the initial concentration of OII in the aqueous solution, implying that the retention of OII on the adsorbent occurs through the available functional groups. Concomitantly, the initial adsorption rate of the same model, which is related to the chemisorption rate, increased, revealing more than one mechanism for the dye uptake on the adsorbent. Similar behavior was revealed by different reports on the removal of OII by adsorption as is the case of the study published by Yardimci et al. [43] who also showed a kinetic behavior described by pseudo-second-order and Elovich models. Wang et al. [49] conducted adsorption experiments of OII on a newly fabricated adsorbent and declared that the process is described by pseudo-second-order kinetics. Jin et al. prepared a surfactant-coated zeolite, put it in contact with OII solution, and nominated the pseudo-second-order model as the best kinetic model for the adsorption process [50]. Luo and his coworkers conducted research on obtaining ion-based nanoparticles synthesized by grape leaf extract and revealed that the OII removal from water fitted the pseudo-second-order kinetic [51].

#### 2.2.4. Effect of Initial OII Concentration and Equilibrium Isotherm

For the study of the initial dye concentration’s influence on the adsorption process, several experiments were conducted at room temperature with 10 mL of OII aqueous solution having concentrations between 10 mg/L and 50 mg/L. The graphical representation of the results given in Figure 4D shows that a removal efficiency of 82.51% and an adsorption capacity of 1.43 mg/g were recorded for an initial concentration of 10 mg/L. At an initial OII concentration of 50 mg/L, the removal efficiency was 78.87%, and the adsorption capacity increased to 6.84 mg/g. This increase may be justified by an enhanced interaction of the adsorbate in higher concentrations with the CSSA hydrogel. Additionally, the presence of OII in a larger amount could be responsible for an increase in the mass transfer of the dye to the adsorbent. These outcomes are comparable with those published by other researchers who attributed the augmentation of the adsorption capacities and the reduction in the removal efficiencies to the increase in the concentration gradient pressure and to the subsequent facilitation of the mass transfer [51,52,53].

In the challenge to clarify the adsorption performance, various notorious isotherm models, namely Langmuir, Freundlich, Jovanovich, Temkin, Toth, Sips, Redlich–Peterson, and Khan were applied to the experimental data. According to the Langmuir model, the adsorbent has a homogeneous surface, each molecule of adsorbate occupies one active site of the adsorbent, and the adsorbate molecules do not interact with each other. The Freundlich isotherm stipulates that the adsorbent has a heterogeneous surface and assumes monolayer adsorption in the case of chemisorption as the main adsorption mechanism and multilayer adsorption when physisorption is the leading adsorption mechanism. The Jovanovich model can be seen as a modified version of the Langmuir isotherm and considers that interactions occur between the adsorbate molecules. The Temkin model hypothesizes that with greater coverage of the adsorbent surface, the adsorption process (defined by a consistent distribution of binding energies) results in a linear reduction in the heat of adsorption for the target molecule, culminating in the maximum bond energy. The Toth model derives from the Langmuir isotherm and describes adsorption in heterogeneous systems that span the entire range of adsorbate concentration. Sips and Redlich–Peterson isotherms derive from both Langmuir and Freundlich models. The first one foresees monolayer adsorption in both homogeneous and heterogeneous conditions overcoming the constraints of the Freundlich isotherm, referring to the increased adsorbate concentrations. The second one denotes the adsorption over an extended adsorbate concentration interval in homogeneous and heterogeneous adsorption systems. The Khan equation reflects the features of Langmuir and Freundlich isotherms, being reduced to the latter one for large values of equilibrium adsorbate concentrations [46,54,55,56].

**Figure 6:**
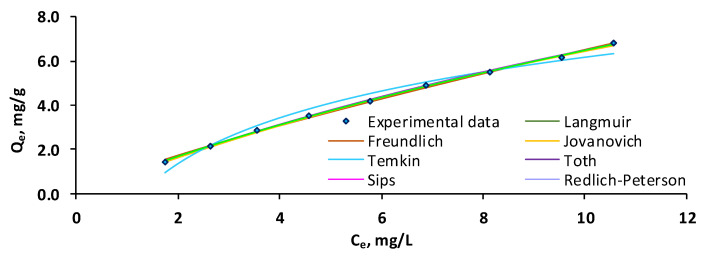
Equilibrium isotherms for the OII adsorption on CSSA hydrogel (the experiments were carried out with 10 mL of OII, at room temperature, without stirring, the working conditions being as follows: OII concentration—variable, *p*H—3, adsorbent dose—0.058 g/L, contact time—1440 min).

From the graphical representation of the tested isotherms (Figure 6), it can be detected that the models overlap each other. This fact could be attributed to the high similarity of the considered isotherms since, according to their assumptions, some of them are variations of the Langmuir and Freundlich equations. A similar good fit with multiple isotherm models was also reported by other research [57,58].

The statistical error functions (Table 2) have reduced and comparable values for all the isotherm models, indicating irrelevant variances between the investigational data and those obtained from the mathematical models.

In consonance with the isotherm parameters, the maximum adsorption capacity calculated with the models is superior to that obtained through experiments, the highest value being recorded for the Sips equation followed by those registered for Langmuir, Toth, and Jovanovich while the best resemblance is observed for the Khan isotherm. Therefore, it can be inferred that the retention of OII from aqueous media is a complex one, being more in line with the Freundlich isotherm than with the Langmuir one. The inverse of the Freundlich constant and the Sips heterogeneity constant, which are both inferior to the unit, along with the negative value the of Toth constant imply a heterogeneous surface of the adsorbent and a favorable multilayer adsorption of OII on the prepared adsorbent [59,60]. The positive value of the Temkin *K_T_* constant indicates an exothermic process, which is in disagreement with the thermodynamic results which will be discussed in the next section.

Many other publications report the use of equilibrium isotherms for the interpretation of the experimental values. Rojas Garcia et al. [61] carried out the adsorption of OII using a metal–organic framework material and established Langmuir as an adequate isotherm to fit the acquired data. They also exposed the fact that the adsorption is favorable based on the Freundlich constant. In the research conducted by Jedynak et al., for the adsorption of OII on micro-mesoporous carbon materials, Langmuir, Freundlich, Langmuir–Freundlich, and Dubinin–Radushkevich models were tested, the first one being declared as suitable for characterizing the process [62]. The information provided by Sonawane et al. in their study directed to the removal of OII through retention on an adsorbent made from *Annona squamosal* shell supports the adequacy of both Langmuir and Freundlich isotherms in describing the process [63]. Aguilar et al. adsorbed OII on *Aloe vera* leaves and indicated the Toth model as appropriate for representing the obtained results [64].

### 2.3. Enhancement of OII Adsorption

Most of the studies dedicated to the removal of unwanted compounds from water by adsorption on various materials usually highlight parameters such as *p*H, adsorbent dosage, time, or initial concentration as being among the most important ones, and different correlations are made between their optimization and the success of the water decontamination. As shown earlier, the adsorbent obtained from the powder of CS entrapped in SA polymeric matrix was able to retain more than 80% of the target dye molecules existing in aqueous solution. In order to evaluate the possibility of obtaining better results, the influence of other three factors that could affect the retention of the water pollutant is discussed herein.

#### 2.3.1. Effect of Stirring

**Figure 7:**
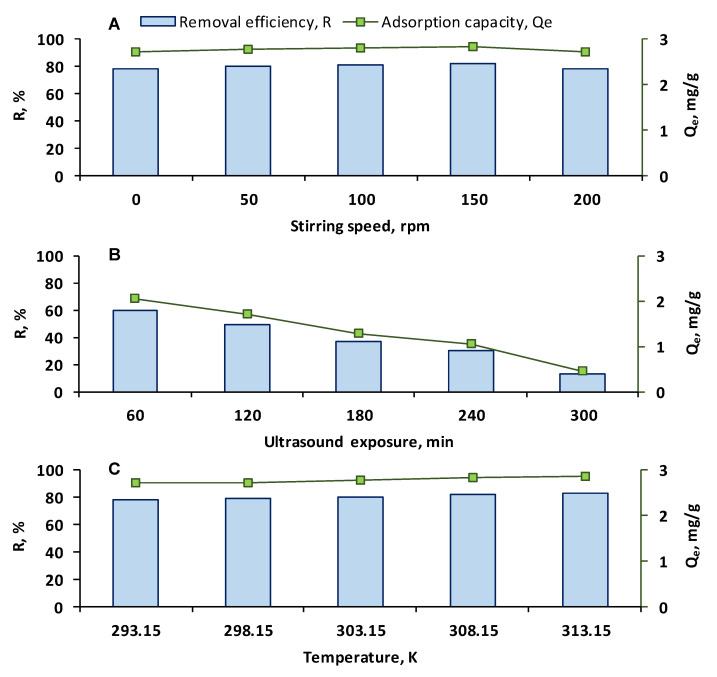
Influence of stirring speed (**A**), ultrasound exposure (**B**), and temperature (**C**) on the removal of OII from aqueous solution through adsorption on CSSA hydrogel (the experiments were carried out with 10 mL of OII, having *p*H 3 and a concentration of 20 mg/L, with an adsorbent dose of 0.058 g/L, for 300 min).

Due to the fact that it could improve the distribution of the adsorbate in the solution and enhance the contact with the adsorbent, stirring could have an impact on the adsorption process. Experimental runs were carried out for 300 min at room temperature with 10 mL of OII having an initial concentration of 20 mg/L, *p*H 3, and an adsorbent dose of 0.058 g/L. Four stirring rates (50, 100, 150, 200 rpm) were applied and the outcomes are presented in Figure 7A.

As noticeable, the removal efficiencies and the adsorption capacities had similar values to those observed in the absence of agitation. The main difference regarded the time required for reaching these results which, for a stirring rate of 150 rpm, was reduced from 300 min to only 180 min. Abdulhameed et al. used a novel biocomposite composed of grafted chitosan–phthalic anhydride/Co_2_O_3_ nanoparticles for the elimination of brilliant green from aquatic systems. They also revealed that the agitation had an accelerating role in the adsorption process [65]. Ritter et al. studied the adsorption of safranine-T on a waste-based zeolite and concluded that an agitation rate of 147 rpm exerts a beneficial impact on dye retention [66]. Kim et al. adsorbed methylene blue and Congo red on pine nut husk biochars modified with hydroxyapatites and showed that satisfactory results were registered under stirring at 150 rpm [67]. On the other hand, in the current study, a slight decrease in the removal efficiency and adsorption capacity was found when the stirring rate was 200 rpm. In their examination of the adsorptive removal of methylene blue by a nanocomposite, Ahmed et al. observed an analog behavior and explained that at higher stirring speed the bonds formed between the dye molecules and the adsorbent could be destroyed, causing a desorption phenomenon [68].

#### 2.3.2. Effect of the Ultrasound Treatment

Another option for improving the adsorption process consists in the use of an ultrasound treatment, the fundamental concept behind this alternative being the fact that the ultrasound waves generate cavitation leading to a better mass transfer of the adsorbate to the adsorbent. Tests were conducted at room temperature with 10 mL of OII having an initial concentration of 20 mg/L, *p*H 3, and an adsorbent dose of 0.058 g/L. The removal efficiencies and the adsorption capacities were calculated after 60, 120, 180, 240, and 300 min of exposure in an ultrasound bath (Figure 7B). The highest values were obtained after 60 min of treatment and were 59.42%, and 2.06 mg/g, respectively (considerably lower than those in the absence of ultrasound) while after 300 min the results were drastically reduced (12.61% and 0.44 mg/g). Provided that the adsorbents are completely different, our outcomes are in disagreement with those published by Munonde et al. who explored the adsorption of reactive red 120 dye on nickel iron layered double hydroxides/activated carbon nanosheets and revealed that the ultrasound treatment was suited for the adsorption process [69]. Saini et al. also emphasize that the ultrasound action intensified the adsorption of several dyes on metal–organic frameworks [70]. In the present research, it must also be noted that, after the first 60 min, the CSSA hydrogel beads maintained their shape and, over time, they started to slowly disintegrate, with small fractions of adsorbent being visible in the aqueous solution. This could be explained by the fact that the cavitation process is too aggressive for the prepared hydrogel, destroying its delicate structure. In pursuance of ultrasound’s advantages, further experiments are required. An option would be to apply the treatment directly on the CS before or after transforming it into powder in view of the subsequent mixing with SA.

#### 2.3.3. Effect of the Temperature and Thermodynamic Analysis

The temperature is a determinant factor in an adsorption process [71,72,73]. Its impact was estimated based on data collected from the experiments executed at 293.15, 298.15, 303.15, 308.15, and 313.15 K with 10 mL of OII having an initial concentration of 20 mg/L, *p*H 3, and an adsorbent dose of 0.058 g/L. The removal efficiencies and the adsorption capacities increased as the temperature rose and ranged from 78.35% at 293.15 K and 82.96% at 313.15 K to 2.72 mg/g at 293.15 K and 2.88 mg/g at 313.15 K (Figure 7C). The observed reduced amelioration of the adsorption process (of only 5.88%) does not completely justify the change in the working temperature by increasing it by 20 K.

**Figure 8:**
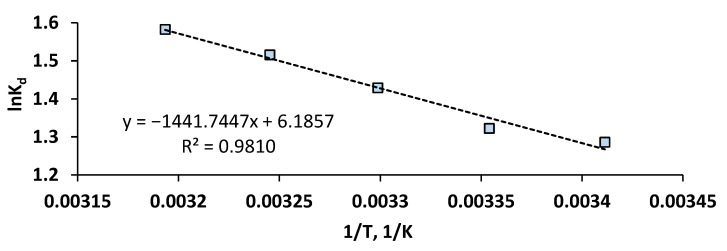
Van ’t Hoff plot for the adsorption of OII on CSSA hydrogel.

The Van ’t Hoff plot (Figure 8), in which *lnK_d_* was represented against *1/T*, served to find the values of *ΔH°/R* as the equation slope and of *ΔS°/R* as its intercept.

The negative values were obtained for ΔG° (−3.09 kJ/mol at 293.15 K, −3.35 kJ/mol at 298.15 K, −3.60 kJ/mol at 303.15 K, −3.86 kJ/mol at 308.15 K, −4.12 kJ/mol at 313.15 K) and the positive values of ΔS° (51.43 J/(mol · K)) and ΔH° (11.99 kJ/mol) imply that the adsorption of OII on the prepared hydrogel is spontaneous, random, and endothermic. Su et al. showed that the removal of dyes such as methylene blue, crystal violet, acid red-73, and acid yellow-17 by retention on porous hydrogel beads was also spontaneous and more promising at higher temperatures [74]. Zhang and his collaborators reveal in their study directed to the adsorption of methylene blue on a gelatin-based functionalized carbon nanotube metal–organic framework adsorbent that the process was characterized as endothermic and random [75]. Similar results were found in other research [8,43]. Concurrently, different conclusions are underlined in other investigations in which dye adsorption was consistent with an exothermic behavior and a diminishment of the system disorder [61] or was considered exothermic in nature but entropy-controlled with greater randomness [76].

### 2.4. Reusability of CSSA Hydrogel

**Figure 9:**
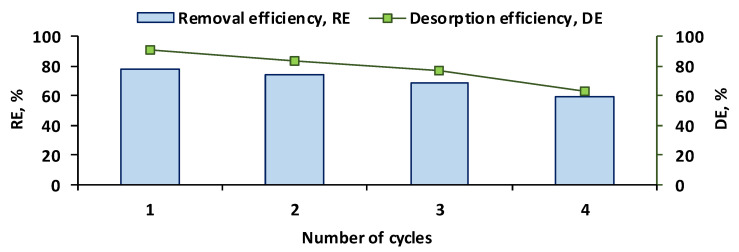
Adsorption–desorption cycles for OII–CSSA hydrogel system (the adsorption experiments were carried out with 10 mL of OII having a concentration of 20 mg/L, and *p*H 3, at room temperature under stirring at 150 rpm, for 300 min; the desorption experiments were carried out for 240 min, with 50 mL of eluent, under stirring at 150 rpm).

After the initial adsorption of OII on the prepared hydrogel, desorption was carried out. Sodium hydroxide solution was found to be the best elution agent since it led to a release of more than 90% of the retained dye after the first contact and more than 60% after the fourth one (Figure 9). The removal efficiency of the target molecule from the aqueous matrix declined considerably after the fourth cycle, reaching only 59.78%. Even though satisfactory results were obtained for the first two cycles of adsorption–desorption, supplementary studies are required in order to optimize the conditions to ensure better prospects for CSSA hydrogel reusability.

### 2.5. Comparison with Other Adsorbents

Table 3 condenses the adsorption capacities of different adsorbents including CSSA and sustains the conclusion that the prepared hydrogel exhibits an encouraging potential for the treatment of wastewater containing OII as a contaminant. The elevated availability and the reduced price of CSSA are some of the supplementary advantages recommending its use for the elimination of target dye pollutants from aqueous matrices.

## 3. Conclusions

The current study was directed to the evaluation of a new green hydrogel prepared from cherry stone powder and sodium alginate as a potential adsorbent of water pollutants. Its performances were evaluated in the presence of Orange II dye as a model molecule. The effect of working parameters (*p*H, adsorbent dosage, time, initial dye concentration) and accelerating factors (stirring rate, ultrasound exposure duration, temperature) was analyzed. A removal efficiency of 82.20% and an adsorption capacity of 6.84 mg/g were registered. Elovich and pseudo-second-order kinetic models fitted the experimental results. The adsorption was described by Freundlich and Khan isotherms and can be considered spontaneous, endothermic, and random. The experiments showed that the obtained hydrogel can be reasonably used for a minimum of two successive cycles.

In light of the aforementioned aspects, it can be inferred that the synthesized product can serve as a promising option for capturing persistent water contaminants.

Future studies will aim firstly at the optimization of the synthesis process in order to use a higher amount of cherry stone powder, secondly the increase in the desorption effectiveness in view of the augmentation of the number of cycles, and thirdly the exploration of the resulting adsorbents’ potential to address an extended range of pollutants existing in real water matrices.

## 4. Materials and Methods

### 4.1. Reagents

Alginic acid sodium salt, Orange II dye, sodium hydroxide, hydrochloric acid, ethanol, sodium chloride, and calcium chloride reagents were used in the experimental program. They were of analytical grade and were bought from Merck (Bucharest, Romania).

All the stock solutions and the required dilutions were prepared with distilled water.

### 4.2. Hydrogel Synthesis

Two precursors, CS and SA, were employed for the synthesis of the new green hydrogel. Collected from the eastern Romanian region, CS was first transformed into powder through a series of treatments including washing, drying (6 h, at 60 °C in a laboratory oven (AirPerformance AP60, Froilabo, Paris, France)), milling (PerkinElmer, Hägersten, Sweden), and sieving (125 µm Filtra Vibracion IRIS FTS-0200 sieve shaker, Filtra Vibracion, Badalona, Spain). At the same time, 50 g of SA aqueous solution (2%) was stirred for 3 h at 300 rpm on a magnetic plate (Auxilab, Beriáin, Spain). Then, 2 g of CS powder was added to the SA solution and the stirring was continued for 3 h. The resultant mixture was dripped into 100 mL of CaCl_2_ (2% in water) which led to obtaining a hydrogel in the form of beads. The resulting CSSA product was stored at 4 °C in closed vessels. It was intensively washed with distilled water before being used for the adsorption process.

### 4.3. Hydrogel Characterization

#### 4.3.1. Physical Properties Analysis

Mean diameter, moisture content, bulk density, and swelling ratio of CSSA hydrogel were the major physical properties analyzed. The mean diameter of CSSA beads was calculated based on the average of the diameter of 10 different hydrogel beads measured with an accuracy of ±0.03 mm with a Carl Roth digital caliper (Bucharest, Romania). For the determination of the moisture content, 10 g of CSSA beads was dried at 60 °C for 6 h in a laboratory oven and weighed after being kept for 30 min in a desiccator filled with calcium chloride. The bulk density (*ρ_b,CSSA_*, g/mL) (Equation (1)) was established as the ratio between the mass of 1 g of dried CSSA beads and the volume occupied by this mass in a 5 mL glass cylinder.

(1) $$\rho_{b,\;CSSA}=\left(\rho_{w}-\rho_{a}\right)\cdot \frac{m_{1}-m_{0}}{\left(m_{2}-m_{0}\right)-\left(m_{3}-m_{1}\right)}+\rho_{a}$$

where: *ρ_w_* = 0.9977 g/cm^3^ is the water density measured at a temperature of 22 °C (Testo 622 thermohygrometer with barometer, Carl Roth, Bucharest, Romania); *ρ_a_* = 0.0019 g/cm^3^ is the air density measured at a temperature of 22 °C, a relative humidity of 38%, and a barometric pressure of 101.991 kPa; *m*_0_ is the weight of a 25 mL glass pycnometer filled with air, g; *m*_1_ is the weight of the pycnometer filled with dried CSSA beads, g; *m*_2_ is the mass of the pycnometer filled with water, g; *m*_3_ is the weight of the pycnometer filled with water and dried CSSA beads, g.

For the swelling ratio (*SR*) (Equation (2)), 0.05 g of dried CSSA (*m_i,CSSA_*) was added to a series of five Erlenmeyer flasks containing 10 mL of distilled water with *p*H 3, 5, 7, 9, 11 (adjusted with HCl 0.1 M or NaOH 0.1 M and measured with an HI 98103 *p*H tester from Hanna Instruments, Bucharest, Romania). The samples were collected and weighed again (*m_f,CSSA_*) after 24 h at room temperature.

(2) $$SR=\frac{m_{f,\;\;CSSA}-m_{i,\;CSSA}}{m_{i,\;CSSA}}\cdot 100$$

#### 4.3.2. SEM Morphology

The CSSA hydrogel beads (dried for 12 h at room temperature) were placed on double-adhesive carbon discs fixed on the specific stubs of a TESCAN MIRA device (TESCAN Orsay Holding, Brno, Czech Republic) equipped with TESCAN Essence software version 1.0.8.0. The parameters used for SEM analysis were as follows: normal secondary electron mode, large field detector, high vacuum, accelerating voltage of 20 keV, beam current of 300 pA, working distance of 35–50 mm, magnification range of 50–500 µm.

#### 4.3.3. FTIR Analysis

The functional groups of the CSSA hydrogel were explored through FTIR examination. An IRSpirit FTIR spectrometer with attenuated total reflectance single-reflection (QATR-S) auxiliary (Shimadzu, Bucharest, Romania) was employed for spectra acquisition in an interval between 4000 and 400 cm^−1^ (50 scans/min) with a resolution of 8 cm^−1^. The QATR-S accessory was cleaned with ethanol between acquisitions.

#### 4.3.4. *p*H_PZC_ Determination

Sodium chloride was used as a background electrolyte for *p*H_PZC_ determination. Aliquots of NaCl 0.1 M (20 mL) with initial *p*H (*p*H_i_) between 2 and 12 were added to a series of Erlenmeyer flasks. Then, 0.5 g of CSSA beads was added. After 24 h at room temperature, the final *p*H (*p*H_f_) was measured and the differences (Δ*p*H) from the initial values were calculated. The *p*H_PZC_ was established at the graphical intersection observed between Δ*p*H and *p*H_i_.

### 4.4. Adsorption Study

A stock solution of OII dye with a concentration of 1000 mg/L was prepared and used for subsequent dilutions. Samples of 10 mL of OII were utilized in all the experiments. A UV1280 spectrophotometer (Shimadzu, Kyoto, Japan) set at 485 nm was employed for recording the absorbance of the calibration curve (1–10 mg/L) and of the OII solutions through the tests.

The effect of parameters such as *p*H (3–11), adsorbent dosage (0.019–0.058 g/L), time (5–1440 min), initial dye concentration (10–50 mg/L), stirring speed (0–200 rpm), ultrasound exposure duration (60–300 min), and temperature (293.15–313.15 K) on the adsorption of OII by the prepared hydrogel was evaluated.

The removal efficiency (*R*, %) and the equilibrium sorption capacity (*Q_e_*) were calculated by applying Equations (3) and (4).

(3) $$R=\frac{C_{i}-C_{e}}{C_{i}}\cdot 100$$

(4) $$Q_{e}=\frac{C_{i}-C_{e}}{m}\cdot V$$

where *C_i_* and *C_e_* are the OII initial and equilibrium concentrations (mg/L); *V* is the OII dye volume (mL); and *m* is the adsorbent amount (g).

The experimental data were fitted with pseudo-first-order, pseudo-second-order, and Elovich kinetic models by using the nonlinear Equations (5)–(7).

(5) $$\mathrm{Pseudo}\text{-}\mathrm{first}\;\mathrm{order}\;Q_{t}=Q_{e}\cdot \left(1-e^{-k_{1}\cdot t}\right)\;$$

(6) $$\;\mathrm{Pseudo}\text{-}\mathrm{second}\;\mathrm{order}\;Q_{t}=\frac{k_{2}\cdot Q_{e}^{2}\cdot t}{\left(1+k_{2}\cdot Q_{e}\cdot t\right)}$$

(7) $$\mathrm{Elovich}\;Q_{t}=\frac{1}{\beta }\cdot \ln\left(\alpha \cdot \beta \cdot t\right)\;$$

where *Q_t_*—concentration on the solid phase at time *t*, mg/g; *Q_e_*—adsorbent capacity at equilibrium, mg/g; *k*_1_—pseudo-first-order constant rate, min^−1^; *t*—contact time, min; *k*_2_—pseudo-second-order constant rate, g/(mg·min); *α*—initial adsorption rate, mg/(g·min); *β*—extent of surface coverage and activation energy for chemisorption, g/mg.

Various isotherm models were also applied to the experimental data. Their equations are given below.

(8) $$\;\mathrm{Langmuir}\;Q_{e}=\frac{Q_{L}\cdot K_{L}\cdot C_{e}}{1+K_{L}\cdot C_{e}}$$

(9) $$\mathrm{Freundlich}\;Q_{e}=K_{F}\cdot C_{e}^{1/n_{F}}$$

(10) $$\mathrm{Jovanovich}\;Q_{e}=Q_{J}\cdot \left(1-e^{K_{J}\cdot C_{e}}\right)$$

(11) $$\;\mathrm{Temkin}\;Q_{e}=\frac{R\cdot T}{B_{T}}\cdot ln\left(K_{T}\cdot C_{e}\right)$$

(12) $$\;\mathrm{Toth}\;Q_{e}=\frac{Q_{To}\cdot C_{e}}{{\left(K_{To}+{C_{e}}^{n_{To}}\right)}^{1/n_{To}}}$$

(13) $$\;\mathrm{Sips}\;Q_{d,t}=\frac{Q_{S}\cdot {\left(K_{S}\cdot C_{e}\right)}^{n_{S}}}{1+{\left(K_{S}\cdot C_{e}\right)}^{n_{S}}}$$

(14) $$\;\mathrm{Redlich}–\mathrm{Peterson}\;Q_{e}=\frac{K_{R}\cdot C_{e}}{1+a_{R}\cdot C_{e}^{n_{R}}}$$

(15) $$\;\mathrm{Khan}\;Q_{e}=\frac{Q_{K}\cdot K_{K}\cdot C_{e}}{{\left(1+K_{K}\cdot C_{e}\right)}^{n_{K}}}$$

where: *Q_e_*—adsorbate concentration on the solid phase at equilibrium, mg/g; *C_e_*—adsorbate concentration on fluid phase at equilibrium, mg/L; *Q_L_*—maximum Langmuir uptake, mg/g; *K_L_*—Langmuir constant, L/mg; *K_F_*—Freundlich constant, (mg/g)(L/mg)^1/*nF*^; *n_F_*—Freundlich constant, dimensionless; *Q_J_*—Jovanovich maximum uptake, mg/g; *K_J_*—Jovanovich constant, L/g; *R—*universal gas constant R = 8.314 J/(mol · K); *T*—temperature, K; *B_T_—*Temkin constant; *K_T_*—Temkin isotherm equilibrium binding constant, L/g; *Q_To_*—Toth maximum uptake, mg/g; *K_To_*—Toth constant, L/mg; *n_To_*—Toth constant, dimensionless; *Q_S_*—Sips maximum uptake, mg/g; *K_S_*—Sips constant, L/mg; *n_S_*—Sips constant, dimensionless; *K_R_*—Redlich–Peterson constant, L/g; *α_R_*—Redlich–Peterson constant, 1/mg; *n_R_*—Redlich–Peterson exponent, dimensionless; *Q_K_*—Khan maximum uptake, mg/g; *K_K_*—Khan constant, L/mg; *n_K_*—Khan exponent, dimensionless.

Equations (16)–(18) were used for establishing the thermodynamic behavior.

(16) $$∆G{}^{\circ}=-R\cdot T\cdot lnK_{d}$$

(17) $$lnK_{d}=\frac{∆S^{{}^{\circ}}}{R}-\frac{∆H^{{}^{\circ}}}{R\cdot T}$$

(18) ∆G°=∆H°−T·∆S°

Δ*G°*—Gibbs free energy, kJ/mol; *R*—gas constant, R = 8.314 J/(mol · K); *T*—temperature, K; *K_d_*—coefficient of distribution determined as ratio of the OII equilibrium concentration in the solid phase (*C_s_*, mg/L) and in the solution (*C_e_*, mg/L); Δ*S°*—change in entropy, J/(mol · K); Δ*H°*—change in enthalpy, kJ/mol.

### 4.5. Adsorbent Recyclability

For the evaluation of the CSSA hydrogel reusability, after the experiments conducted for 300 min at room temperature with 10 mL of OII solution having a concentration of 20 mg/L and *p*H 3, the adsorbent was recovered and subjected to regeneration. The process was performed for 240 min, under stirring at 150 rpm, with 50 mL of water, ethanol, sodium hydroxide 0.05 M, and hydrochloric acid 0.05 M. The regenerated adsorbent was reused for OII retention.

The desorption efficiency (*D*, *%*) was expressed using Equation (19).

(19) $$D=\frac{C_{des.}}{C_{ads.}}\cdot 100$$

where *C_des._* is the OII final concentration in the desorbent (mg/L); *C_ads._* is the OII final concentration after adsorption (mg/L).

### 4.6. Statistical Analysis

All the experiments were conducted in triplicate and the results averaged. RMSE, χ^2^, MPSD, HYBRID, R^2^, AICc, and BIC (Equations (20)–(26)) were the statistical error functions applied for verifying the fitting of the acquired data to the kinetic and isotherm mathematical models.

(20) $$\mathrm{RMSE}\;RMSE=\sqrt{\frac{\sum_{i=1}^{n}{\left(Q_{e,\;exp}-Q_{e,\;pred}\right)}^{2}}{N-p}}$$

(21) $$\chi^{2}\;\chi^{2}=\sum_{i=1}^{n}\frac{{\left(Q_{e,\;exp}-Q_{e,\;pred}\right)}^{2}}{Q_{e,\;pred}}\;$$

(22) $$\mathrm{MPSD}\;MPSD=100\cdot \sqrt{\frac{1}{N-p}\cdot \sum_{i=1}^{n}{\left(\frac{Q_{e,\;exp}-Q_{e,\;pred}}{Q_{e,\;exp}}\right)}^{2}}$$

(23) $$\;\mathrm{HYBRID}\;HYBRID=\frac{100}{N-p}\cdot \sum_{i=1}^{n}\left[\frac{{\left(Q_{e,\;exp}-Q_{e,\;pred}\right)}^{2}}{Q_{e,\;exp}}\right]$$

(24) $$\;R^{2}\;R^{2}=\frac{\sum_{i=1}^{n}{\left(Q_{e,\;pred}-\bar{Q_{e,\;exp}}\right)}^{2}}{\sum_{i=1}^{n}{\left(Q_{e,\;pred}-\bar{Q_{e,\;exp}}\right)}^{2}+\sum_{i=1}^{n}{\left(Q_{e,\;pred}-Q_{e,\;exp}\right)}^{2}}$$

(25) $$\;\mathrm{AICc}\;AIC_{c}=2\cdot p+N\cdot ln\left(\frac{SSE}{N}\right)+\frac{2\cdot p\cdot \left(p+1\right)}{N-p-1}$$

(26) $$\;\mathrm{BIC}\;BIC=2\cdot p\cdot \ln\left(lnN\right)+N\cdot ln\left(\frac{SSE}{N}\right)$$

where: *N*—number of experimental data points; *p*—number of the mathematical model parameters; *Q_e,exp_*—experimental adsorption capacity; *Q_e,pred_*—predicted adsorption capacity.

## Figures and Tables

**Table 1:** Kinetic parameters and error functions for the adsorption of OII on CSSA hydrogel.

Kinetic Model	Parameters	Initial Orange II Concentration, mg/L								
		10	15	20	25	30	35	40	45	50
Experimental data	Q_e_	1.43	2.14	2.85	3.54	4.19	4.88	5.52	6.14	6.84
Pseudo-first order	Q_e_	1.31	1.95	2.57	3.20	3.83	4.40	5.02	5.57	6.23
	*k*_1_	0.13	0.14	0.14	0.15	0.03	0.18	0.20	0.23	0.25
	RMSE	0.1206	0.1798	0.2393	0.2672	0.2963	0.3682	0.3773	0.4053	0.4300
	χ^2^	0.2407	0.3517	0.4650	0.4636	0.4724	0.6117	0.5542	0.5608	0.5576
	MPSD	11.8347	11.6868	11.6428	10.4064	9.5147	10.1266	8.9427	8.4691	7.9640
	HYBRID	1.4882	2.1998	2.9111	2.9107	2.9593	3.9191	3.5522	3.6185	3.6131
	R^2^	0.5881	0.5234	0.5363	0.5686	0.5439	0.4595	0.4631	0.4248	0.3920
	AICc	−71.8714	−57.5053	−47.2108	−43.2395	−39.5217	−31.6964	−30.8145	−28.2391	−26.113
	BIC	−67.4669	−53.1008	−42.8063	−38.8350	−35.1172	−27.2919	−26.4100	−23.8346	−21.7086
Pseudo-second order	Q_e_	1.37	2.03	2.68	3.33	3.96	4.56	5.18	5.74	6.41
	*k*_2_	0.14	0.11	0.08	0.07	0.07	0.07	0.07	0.07	0.07
	RMSE	0.0727	0.1114	0.1507	0.1592	0.1756	0.2350	0.2355	0.2628	0.2800
	χ^2^	0.0923	0.1432	0.1927	0.1709	0.1716	0.2601	0.2238	0.2430	0.2447
	MPSD	7.3210	7.3870	7.4408	6.2690	5.6744	6.4916	5.5757	5.4833	5.1766
	HYBRID	0.5541	0.8610	1.1696	1.0427	1.044	1.6019	1.3812	1.5180	1.5287
	R^2^	0.8500	0.8168	0.8161	0.8468	0.8397	0.7799	0.79083	0.7582	0.7421
	AICc	−90.0581	−74.7180	−63.8609	−61.8771	−58.3445	−47.867	−47.7808	−43.8415	−41.5543
	BIC	−86.6104	−70.3135	−59.4564	−57.4726	−53.9400	−43.4625	−43.3758	−39.4370	−37.1498
Elovich	*α*	27.17	88.80	86.40	245.76	1034.04	1267.97	2845.75	9772.13	70,547.83
	*β*	8.25	5.95	4.39	0.16	3.53	3.08	0.11	0.161	2.79
	RMSE	0.0341	0.0463	0.0151	0.0672	0.077	0.0742	0.0730	0.0779	0.0714
	χ^2^	0.0152	0.0189	0.0172	0.0251	0.0283	0.0217	0.0185	0.0190	0.0144
	MPSD	2.7604	2.4776	2.0565	2.2698	2.1991	1.7459	1.5061	1.4473	1.1979
	HYBRID	0.0984	0.1210	0.1106	0.1596	0.1790	0.1369	0.1163	0.1193	0.0905
	R^2^	0.9670	0.9683	0.9788	0.9726	0.9687	0.9780	0.9798	0.9787	0.9832
	AICc	−111.3500	−106.303	−102.775	−92.8872	−87.7506	−89.3567	−89.9075	−87.6105	−90.7439
	BIC	−112.9453	−101.8982	−98.3706	−88.4827	−83.3461	−84.9522	−85.5030	−83.2060	−86.3394

**Table 2:** Equilibrium isotherm parameters and error functions for the adsorption of OII on CSSA hydrogel.

Isotherm	Parameters		RMSE	χ^2^	MPSD	HYBRID	R^2^	AICc	BIC
Langmuir	*Q_L_*	23.35	0.0682	0.0066	1.6178	0.0958	0.9987	−37.7715	−43.9798
	*K_L_*	0.04							
Freundlich	*K_F_*	0.994	0.0906	0.0213	4.1615	0.3232	0.9978	−32.6757	−38.8841
	*n_F_*	1.23							
Jovanovich	*Q_J_*	13.48	0.0715	0.0070	1.5599	0.1006	0.9986	−36.9298	−43.1382
	*K_J_*	0.07							
Temkin	*B_T_*	375.76	0.3329	0.3340	13.6891	3.8074	0.9713	−9.2599	−15.4682
	*K_T_*	0.79							
Toth	*Q_To_*	23.35	0.0737	0.0066	1.7475	0.1118	0.9987	−27.7715	−41.7826
	*K_To_*	0.04							
	*n_To_*	−29.79							
Sips	*Q_S_*	26.17	0.0731	0.0072	2.0172	0.1224	0.9988	−27.9340	−41.9451
	*K_S_*	0.032							
	*n_S_*	0.98							
Redlich–Peterson	*K_R_*	0.94	0.0719	0.0073	2.0961	0.1237	0.9988	−28.2259	−42.2370
	*α_R_*	0.07							
	*n_R_*	0.82							
Khan	*Q_K_*	8.03	0.0708	0.0072	2.1125	0.1224	0.9988	−28.4986	−42.5097
	*K_K_*	0.12							
	*n_K_*	0.47							

**Table 3:** Adsorption capacities of various adsorbents against OII.

Adsorbent	Adsorption Capacity, mg/g	Reference
Granular bentonite–sawdust–corncob	12	[41]
Hexadecyltrimethylammonium bromide-coated natural zeolite	38.96 8.13	[50]
Custard apple fruit shell	2.14	[63]
Aloe vera leaves	8.55	[64]
Modified mussel shell powder	65.43	[77]
Wheat straw	6	[78]
CSSA	6.83	Current study

## Data Availability

Data are contained within the article.
